# Marek’s disease herpesvirus serotype 1 in broiler breeder and layer chickens in Malaysia

**DOI:** 10.14202/vetworld.2019.472-476

**Published:** 2019-03-30

**Authors:** Iryanti Othman, Erkihun Aklilu

**Affiliations:** Department of Paraclinical Studies, Faculty of Veterinary Medicine, Universiti Malaysia Kelantan, 16100 Pengkalan Chepa, Kota Bharu Kelantan

**Keywords:** lymphoproliferative diseases, Marek’s disease virus-1, Marek’s disease, molecular detection, poultry diseases

## Abstract

**Aim:**

This study aimed to investigate the occurrence of Marek’s disease (MD) in five poultry farms in Malaysia using postmortem examination, histopathology, and polymerase chain reaction (PCR).

**Materials and Methods:**

Tissue samples were collected from 24 broiler breeder chickens from four commercial broiler breeder farms and six layer chickens from one layer farm. Gross and histopathological examinations and PCR amplification of the gene encoding for avian MD herpesvirus (MDV-1) were conducted.

**Results:**

Gross pathological changes including hepatomegaly, splenomegaly, lymphomatous lesion at the mesentery, oviduct atrophy, and follicular atresia with lymphomatous were observed, whereas diffuse multifocal whitish infiltration of the spleen, neoplastic infiltration in the liver, intrafollicular lymphoid infiltration of the bursa of Fabricius, and lymphomatous tumor at the mesentery were seen on histopathological examinations. Confirmation by PCR showed that a total of 16 (53.33%) samples were positive for avian MDV-1. Although the outbreak involved a much larger number of birds in the respective farms, our investigation was limited based on resource and time frame allocated for the study.

**Conclusion:**

The findings from this study help in emphasizing the potential threats of MDV to the poultry industry globally, in general, and in Malaysia, in particular. As the scope of the current study is limited, future studies focusing on MDV pathogenesis, typing, and causes of vaccine failures are recommended.

## Introduction

Marek’s disease (MD) is a lymphoproliferative disease caused by MD herpesvirus (MDV), a cell-associated virus which is a member of the Herpesviridae family. There are three serotypes of MDV that differ in their virulence, ability to induce T-cell lymphomas, and antigenic properties. Among the three serotypes, the oncogenic one is serotype 1 MDV [1,2]. The disease is characterized by multiple T-cell lymphoma formation in the viscera, muscle, and skin as well as lesions in peripheral nervous tissues [3]. It occurs in chickens of 3-4 weeks of age or older and is the most common in chickens between 12 and 30 weeks of age [4].

Birds get infected by lateral transmission; direct or indirect contact between birds, inhalation of infected dust containing contaminated dander, and following a complex life cycle, the virus is shed from the feather follicle of infected birds [5]. Depending on the strain of MDV-1, lymphomatosis can occur, especially in the ovary, liver, spleen, kidneys, lungs, heart, proventriculus, and skin. Tumor lesions occur due to the malignant transformation of the lymphocytes causing T-cell lymphomas in chickens [4]. There are several diagnostic methods for MD, and the use of polymerase chain reaction (PCR) was found to be rapid and more specific for the detection of MDV-1. PCR is also a highly sensitive test in detecting MDV-1 and enables differentiation of oncogenic and non-oncogenic strains of serotype 1 MDV and MDV vaccine strains of serotypes 2 and 3 [6,7]. Detection of MDV in clinically affected and apparently healthy birds is helpful to know the presence of virus in poultry flock and institute appropriate prevention and control measures against it.

MD is a major economic risk for poultry farms as it occurs in almost all commercial chicken farms and causes significant economic loss with an estimated annual loss up to the US $2 billion worldwide [8,9]. Mortality rarely exceeds 10-15% and can occur over a few weeks or many months [10]. Due to the unpredictability of outbreaks and the possibility of vaccination failure as a consequence of the evolution of more virulent strains of MDV, MD remains a major concern for the poultry industry [11]. In Malaysia, the poultry industry has a significant economic contribution among the agricultural sectors and chicken meat is one of the most consumed foods among the urban and rural communities in the country [12]. Although MD is known to be found in most poultry farms worldwide, the status of this disease in Malaysia is poorly communicated.

This study aimed to detect the presence of avian herpesvirus that causes MD in broiler breeder and layer farms in Malaysia using PCR and to optimize the PCR procedures for detecting MDV-1.

## Materials and Methods

### Ethical approval

This study was conducted after getting approval from the Animal Ethics Committee at the Faculty of Veterinary Medicine, Universiti Malaysia Kelantan.

### Postmortem examination and sample collection

Samples of 30 dead chickens from five farms with a history of suspected MD outbreak were collected. The samples for MDV detection were collected from 24 broiler breeder chickens from four commercial broiler breeder farms and six layer chickens from one layer farm located at Perak and Penang, Malaysia. Samples were taken from the chicken with a history of mortality and postmortem lesions suggestive of MD. The samples taken were comprised of the liver, spleen, and ovary for each chicken. All the chicken’s carcasses were thoroughly inspected, and gross pathological conditions were identified and recorded. Meanwhile, any tissues with gross pathological changes suggestive of MD were collected for further analysis.

### Histopathological examination

Samples of different organs were kept in 10% formalin until fixation. Then, tissues were routinely processed into paraffin blocks, sectioned at 5 μm, deparaffinized, stained with H and E, and finally examined under a light microscope.

### Sample preparation and DNA extraction

The pooled samples comprised of the liver, spleen, and ovary for each chicken were grounded in a mortar using sterile mortars and pestles with sterile sand as described by Abdel-Latif and Khalafalla [13] with minor modifications. 2 ml of phosphate buffer saline (PBS) was added to the pooled samples and the organs were grounded using mortar and pestle and adding sterile sand, and the grinding was done until the tissue samples were consistent, finely ground to achieve a homogenized paste. An additional 2-ml PBS was gradually added until a 10% suspension was obtained. Then, the suspension was centrifuged at 3000 rpm for 15 min. The supernatant was collected into sterile bottles and treated with 4 ml of penicillin-streptomycin (10,000 IU/ml). The supernatant was stored at −20°C until used. Extraction of the genomic DNA from the samples and the positive control (commercial vaccine Rispens CVI 988) was conducted using Promega SV Genomic DNA Purification Kit (Promega, USA) per the procedures recommended by the manufacturer. The positive control, commercial vaccine Rispens CVI 988, was kindly supplied by Rhone Ma (M) Sdn Bhd.

### Amplification of serotype 1 MDV-specific gene

The primers used to amplify the gene of serotype 1 MDV-1 were designed as a sequence published by Handberg *et al*. [14]. The PCR was performed by amplifying the conserved ICP4 gallid herpesvirus-2 gene using the primer sequences, MDV-1.1 5’ GGATCGCCCACCACGATTACTACC 3’ and MDV-1.8 5’ ACT GCC TCA CAC AAC CTC ATC TCC 3’. The PCR reaction was performed in a final volume of 20 µl including 4 µl of nuclease water, 10 µl of 1x MasterMix Vivantis^®^, 2 µl of template, and 2 µl of each 1 µM of forward and reverse primer. The amplification was done using MyCycler^™^ Thermal Cycler (Bio-Rad, USA). A protocol by Abdel-Latif and Khalafalla [13] was used with minor modifications. Briefly, the protocol was set as follows: Initial denaturation at 95°C for 1 min, followed by 31 cycles of denaturation at 95°C for 1 min, annealing at 55°C for 10 s, extension at 72°C for 2 min for each cycle, and a final extension at 72°C for 4 min. The PCR products were analyzed by separating the products by gel electrophoresis in 1.5% agarose gel containing Midori Green. Finally, the gel was analyzed using GelDoc^™^ EZ Imager (Bio-Rad, USA).

## Results

### Postmortem findings

The postmortem findings observed in the examined 30 dead chickens include hepatomegaly and diffuse multifocal whitish infiltration of the liver, splenomegaly and diffuse multifocal whitish infiltration of the spleen, emaciated with crooked keel bone, raised focal whitish infiltration of single nodular tumor in the myocardium, lymphomatous lesion at the mesentery, oviduct atrophy and follicular atresia with lymphomatous lesion, and hemorrhagic proventriculitis. However, there were no lesions seen in the brain and peripheral nerves.

### Histopathological findings

Histopathology result of the liver shows loss of architecture of the liver parenchyma and pleomorphic populations of neoplastic lymphoreticular cells with pyknotic nuclei. Higher magnification shows neoplastic infiltration in the liver which is pleomorphic and the cell nuclei tend to be karyorrhexis. There was also an intrafollicular lymphoid infiltration of the bursa of Fabricius. Lymphomatous tumor at the mesentery shows that the tumor was encapsulated with mononuclear cell infiltrations with the infiltration of lymphocytic cells. There are no lesions seen in the brain and the sciatic nerve.

### PCR amplification

In this research, 30 samples were tested for the detection of avian MDV-1 in broiler breeder and layer chickens using PCR. A total of 16 (53.33%) samples were positive for avian MDV-1, while 14 of the 30 samples were found to be negative for avian MDV-1. Among the five farms tested, four were positive for avian MDV-1. Of these, 75% (12/16) of the positive samples were from broiler breeder farms, while the remaining 25% (4/16) were from the layer farm.

## Discussion

In this study, MDV-1 was detected in 53.33% (16/30) of the tested chicken comprising 12 broiler breeders and four layer chickens. The overall prevalence recorded in this study is higher in comparison to some of the MD prevalence studies conducted in poultry farms elsewhere. In a study reported by Suresh *et al*. [15], serotype 1 MDV was detected in 20% of the samples that they collected from 15 poultry farms. However, other studies reported higher rates of prevalence of MD in poultry farms. In a study conducted by Handberg *et al*. [14], MDV-1 was detected by PCR in spleen tissue from all the flocks at rates varying between 10% and 70% and in feather tip extracts at rates varying between 60% and 100%. In the acute form, which is usually manifested with lymphoma formation in the viscera, 10-30% and up to 70% disease incidence and outbreaks, respectively, can occur [4]. In the current study, MDV-1 was detected despite the fact that all the chicken were vaccinated for MD earlier. Failure of vaccine protection is a common incidence that often hampers the control and prevention efforts. A similar finding was reported by Handberg *et al*. [14] in which they reported that vaccination with either MDV-1 or MDV-3 vaccine did not protect the layer flocks of chicken against MD. It was also reported that vaccinated chickens may shed virulent virus into the environment and because of its immunosuppressive abilities, MDV-1has evolved to become more competent in immune system disarmament or evasion [16]. However, the reason why the chickens were not protected against MD although there were vaccinated was not investigated in the current study. Vaccination of birds is mostly done by administering the vaccine by in ovo route to the developing embryo or into 1-day-old birds immediately after hatching. Although it reduces the rate of progression from MDV latency to lymphomas that kill the bird, it has been reported that vaccination does not prevent infection with MDV [17]. To understand the main reasons for the failure of MD vaccines to protect chicken, a further study addressing vaccine efficacy and typing and characterization of MDV from poultry farms is being considered.

Several methods of the diagnosis of MD have been used, and each method has its own advantages and disadvantages. Such methods include virus isolation, antigen detection, PCR, and serological tests such as agar gel immunodiffusion, direct fluorescent antibody test (FAT), and indirect FAT. The use of PCR in studies of MDV has been widely reported, and the technique has been used for detection, characterization [14,15], and quantification of MDV genome copies [18-20]. In addition, PCR has been used to quantitate virus load in tissues [19,21,22]. Furthermore, PCR has been used to differentially detect MDV and HVT in the blood or feather tips [19,23] and to differentiate oncogenic and non-oncogenic strains of serotype 1 MDV and MDV vaccine strains of serotypes 2 and 3 [6,14,24]. In the current study, PCR was used to detect MDV-1 from tissue samples collected from chickens suspected of having MD.

Infection with MD naturally occurs through aerosol route as a result of inhalation of infected dust from the poultry houses [5]. Following inhalation, the virus moves to the lymphoid organs where it remains as cell associated. The pathogenesis of MDV includes an early cytolytic phase in B cells followed by latency and neoplastic phase in T cells that result in clinical signs such as paralysis and immune suppression. The virus also infects the feather follicles and leads to the release of infectious cell-free particles [3]. This release might serve as a source of infection for healthy birds and possibly result in a wider spread of MDV in the flock. The disease occurs at 3-4 weeks of age or older and is the most common between 12 and 30 weeks of age. It is associated with several distinctive pathological syndromes including lymphoproliferative syndromes which are the most frequent and are of most practical significance [4]. In addition to the problems, it causes in poultry, the oncogenic MDV has been implicated as a potential source of infection to humans who come in contact with chickens having the virus. Studies have indicated that avian oncogenic MDVs were detected in human sera [25,26]. MD has been causing significant economic losses to the poultry industry as a result of the mortality and morbidity that it causes in poultry, particularly, chicken. These economic losses have been estimated to be about 2 billion USD annually [9]. The economic loss due to MD is further increased as the current vaccination-based control and prevention strategy is being undermined due to the continued enhancement of the pathogen virulence. The threats of MDV to the poultry industry are further compounded by the fact that the oncogenic avian herpesvirus is becoming more virulent and evading control by available vaccines, thereby posing an increasing challenge for poultry producers [27].

## Conclusion

In this study, it is believed that the spread of MDV might have been facilitated due to the presence of infected chicken, poor management, and failure of vaccine to render protection to the chicken. In this study, all the chickens investigated were dead birds with postmortem findings suggestive of MD. Although the outbreak involved a much larger number of birds in the respective farms, our investigation was limited based on resource and time frame allocated for the study. However, the findings from this study help in emphasizing the potential threats of MDV to the poultry industry globally, in general, and in Malaysia, in particular. Our findings indicate that there are no nerve lesions in the study subjects confirmed to be MD positive. Hence, further investigation will focus on the characterization of representative MDV associated with MD without nerve lesions. As the scope of the current study is limited, future studies focusing on MDV pathogenesis, typing, and investigations into the causes of vaccine failures are recommended.

## Authors’ Contributions

IO conducted the fieldwork and laboratory activities, and EA designed the research project, supervised and guided the research activities, analyzed and interpreted the laboratory results, and wrote the manuscript. Both authors read and approved the final manuscript.
